# Dementia across the Lifespan and around the Globe—Pathophysiology, Prevention, Treatment, and Societal Impact: A Call for Papers

**DOI:** 10.1371/journal.pmed.1002117

**Published:** 2016-08-30

**Authors:** The PLOS Medicine Editors

**Affiliations:** The *PLOS Medicine* Editors are Clare Garvey, Thomas McBride, Linda Nevin, Larry Peiperl, Amy Ross, Paul Simpson, and Richard Turner

## Abstract

In this months editorial, the *PLOS Medicine* Editors announce an upcoming Special Issue and call for papers, with Guest Editors Carol Brayne and Bruce Miller, on dementia across the lifespan and around the globe.

As part of our continued commitment to disseminating open-access research and commentary on the world's most pressing health challenges, *PLOS Medicine* is now inviting submissions of research papers to be considered for publication in a special issue on the pathophysiology, prevention, treatment, and societal impact of dementia. This special issue, planned for publication in March, 2017, will be guest edited by Dr. Carol Brayne, Director of the Cambridge Institute for Public Health at Cambridge University, and Dr. Bruce Miller, Director of the Aging and Memory Center at the University of California, San Francisco, and will also include commissioned Editorials and Perspectives written by leaders in the field.

Alzheimer’s Disease International (ADI) estimates that in 2015 there were 46.8 million cases of dementia worldwide, with a total cost exceeding US$ 818 billion for the year [1]. Although confirming the specific type of dementia in a given individual is notoriously difficult [2,3], Alzheimer’s disease is generally accepted as the leading pathological cause, followed by vascular dementia, Lewy body, and frontotemporal dementia [4]. With an increasing number of adults living to older ages, ADI projects that there will be over 131.5 million people living with dementia by 2050. In addition to this steep increase in cases, a shift in burden from the richest to poorest countries is anticipated; the percentage of people with dementia living in low or middle income countries is expected to increase from 58% in 2010 to 71% in 2050 [5].

These trends emphasize the need for a greater understanding of dementia from a global perspective, not only in the literal sense—the etiology, epidemiology, and impact of the disease around the world—but also encompassing multiple dimensions of disease: genetic and environmental risk factors across an individual’s lifespan; the numerous pathophysiological and clinical entities that fall under the “dementia” umbrella; actions to prevent, delay, treat, and care for the disease; and the levels across which those actions can be implemented (individual, household, community, or society).

Accordingly, for this special issue we are inviting submissions across five general areas covering a broad range of dementia research:

**Genetic and Environmental Risk Factors for Dementia:** Research investigating how dementia risk varies across geographical settings, reflecting isolated genetic populations or societal differences; how diagnoses or demographic patterns have changed from generation to generation; and how risks for dementia differ according to age at diagnosis.

**Variation in Specific Types of Dementia:** Research on how types of dementia are best characterized according to cause and clinical presentation, with special consideration of early- versus late-onset variations.

**Interventions to Prevent Dementia or Slow Its Progression:** Evidence for or against interventions that may reduce risk of dementia or may delay the progression from mild cognitive impairment to severe dementia. These include pharmaceutical, nutritional, physiological, cognitive, and psychosocial approaches. Similarly, the scope of the special issue will include research on how best to implement effective interventions at individual, household, community, government, and societal levels.

**Advances in Diagnosis and Early Detection/Screening:** Research on early detection of dementia—as a prerequisite for studying early pathogenesis and progression, and for evaluating potential early interventions—including strategies for screening and predictive biomarkers with relevance to clinical care or public health.

**Current and Projected Societal Impact of Dementia, Public Policy Implications, and Ethics of Dementia Screening and Care around the World:** Research on the comparative impact of dementia on families, communities, health systems, and economies—for example, in urban versus rural settings, or in high-income versus low-income countries—and effective and ethical policies to deal with the increasing burden of dementia across settings.

**The deadline for submissions is Friday, September 30, 2016.**

Please submit your manuscript through our submissions site at the following address: http://journals.plos.org/plosmedicine/s/submit-now.

Authors are not required to send a pre-submission inquiry when submitting a manuscript for the Special Issue on Dementia. Please indicate in your cover letter that you would like the full manuscript to be considered for the special issue. Authors of submissions that represent sound science but are not selected for the issue itself may be offered transfer of the manuscript to *PLOS ONE*, with inclusion in an overall Collection on Dementia that will include papers published in both journals. If you would like to enquire about the suitability of a manuscript for consideration in the special issue, please e-mail plosmedicine@plos.org.

We look forward to your research on this increasing burden upon lives and communities worldwide.
